# Supplementary figures and images for: Disruption of the novel nested gene Aff3ir mediates disturbed flow-induced atherosclerosis in mice
Source: eLife. 2025 May 2;13:RP103413. doi: 10.7554/eLife.103413 (PMC12048156; doi:10.7554/eLife.103413)

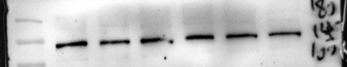

Supplement: Figure 1—source data 2. [file elife-103413-fig1-data2.zip › Figure 1–Source Data 2/AFF3.tif]

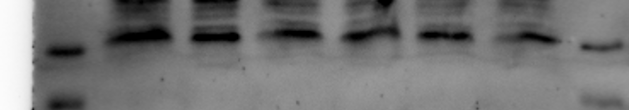

Supplement: Figure 1—source data 2. [file elife-103413-fig1-data2.zip › Figure 1–Source Data 2/AFF3ir-ORF1.tif]

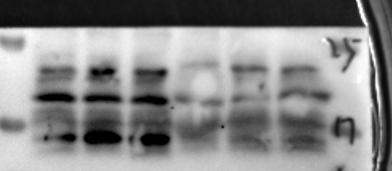

Supplement: Figure 1—source data 2. [file elife-103413-fig1-data2.zip › Figure 1–Source Data 2/AFF3ir-ORF2.tif]

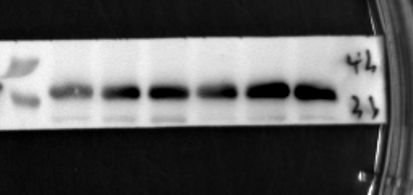

Supplement: Figure 1—source data 2. [file elife-103413-fig1-data2.zip › Figure 1–Source Data 2/GAP.tif]

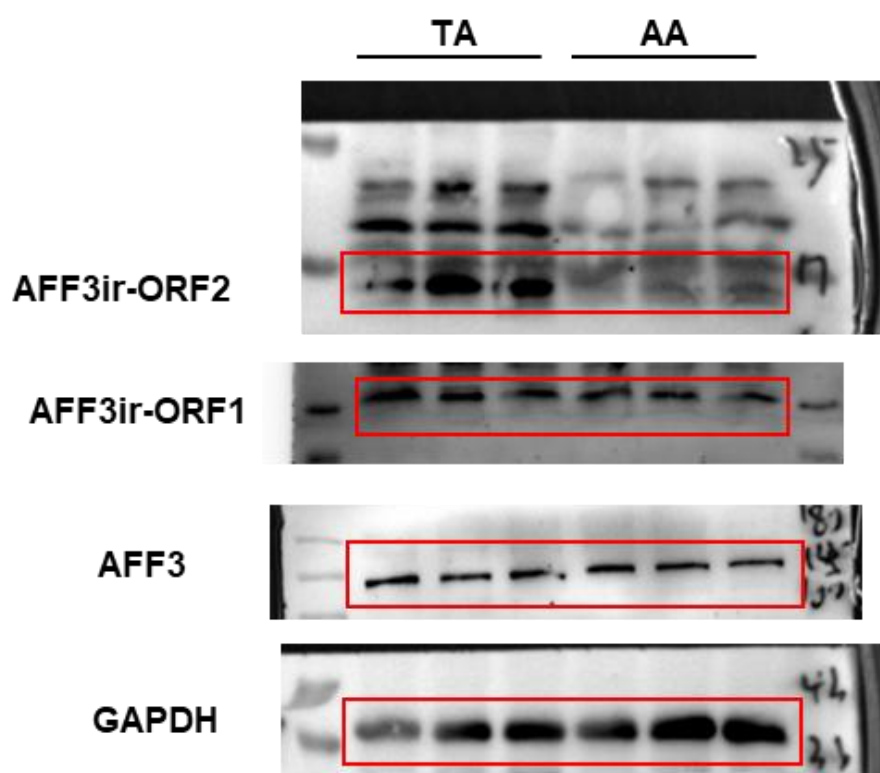

Supplement: Figure 1—source data 3. [file elife-103413-fig1-data3.zip › Figure 1–Source Data 3.pdf]

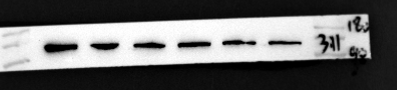

Supplement: Figure 1—source data 4. [file elife-103413-fig1-data4.zip › Figure 1–Source Data 4/AFF3.tif]

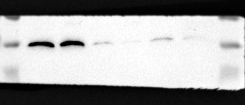

Supplement: Figure 1—source data 4. [file elife-103413-fig1-data4.zip › Figure 1–Source Data 4/AFFir-ORF2.tif]

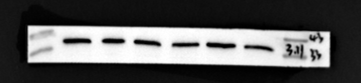

Supplement: Figure 1—source data 4. [file elife-103413-fig1-data4.zip › Figure 1–Source Data 4/GAP.tif]

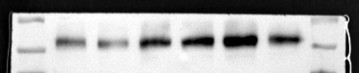

Supplement: Figure 1—source data 4. [file elife-103413-fig1-data4.zip › Figure 1–Source Data 4/ICAM-1.jpg]

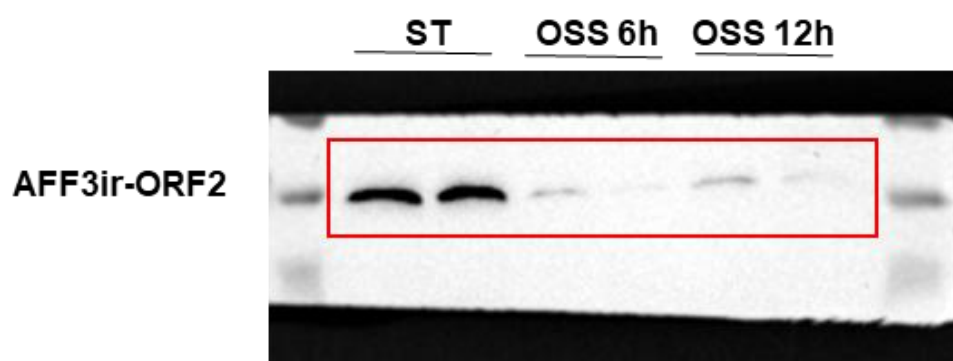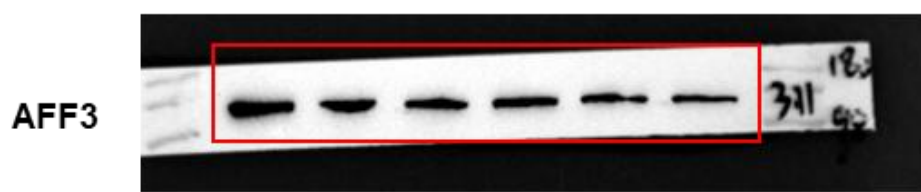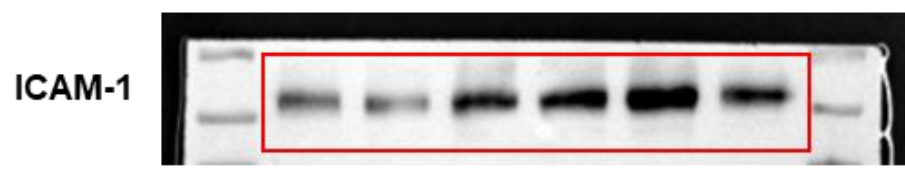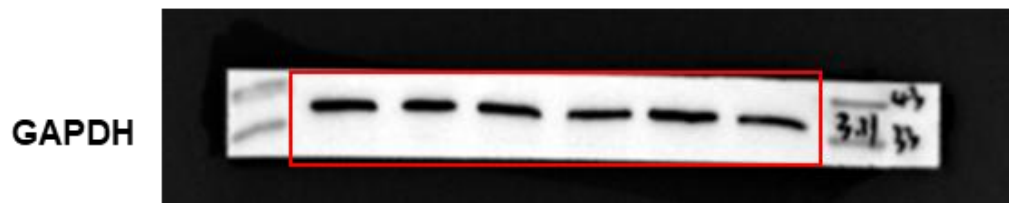

Supplement: Figure 1—source data 5. [file elife-103413-fig1-data5.zip › Figure 1–Source Data 5.pdf]

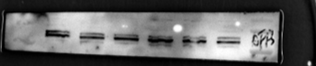

Supplement: Figure 1—figure supplement 1—source data 2. [file elife-103413-fig1-figsupp1-data2.zip › Figure 1-Figure Supplement 1-Source Data 2/AFF3.tif]

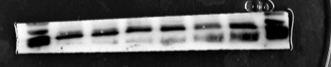

Supplement: Figure 1—figure supplement 1—source data 2. [file elife-103413-fig1-figsupp1-data2.zip › Figure 1-Figure Supplement 1-Source Data 2/AFF3ir-ORF1.tif]

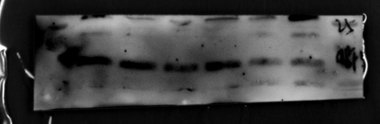

Supplement: Figure 1—figure supplement 1—source data 2. [file elife-103413-fig1-figsupp1-data2.zip › Figure 1-Figure Supplement 1-Source Data 2/AFF3ir-ORF2.tif]

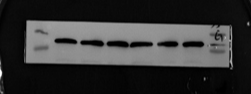

Supplement: Figure 1—figure supplement 1—source data 2. [file elife-103413-fig1-figsupp1-data2.zip › Figure 1-Figure Supplement 1-Source Data 2/GAP.tif]

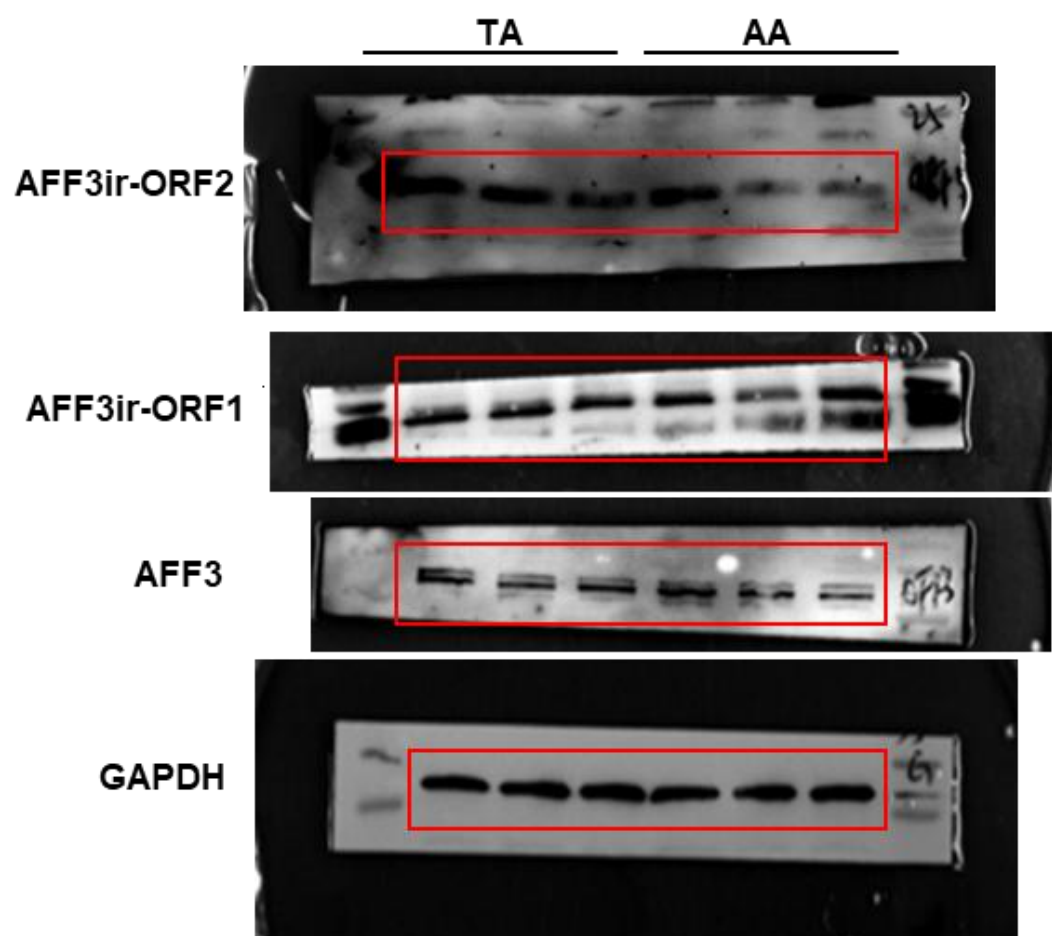

Supplement: Figure 1—figure supplement 1—source data 3. [file elife-103413-fig1-figsupp1-data3.zip › Figure 1-Figure Supplement 1-Source Data 3.pdf]

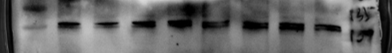

Supplement: Figure 2—source data 2. [file elife-103413-fig2-data2.zip › Figure 2–Source Data 2/AFF3.tif]

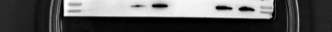

Supplement: Figure 2—source data 2. [file elife-103413-fig2-data2.zip › Figure 2–Source Data 2/FLAG.tif]

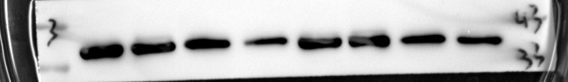

Supplement: Figure 2—source data 2. [file elife-103413-fig2-data2.zip › Figure 2–Source Data 2/GAP.tif]

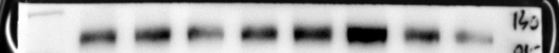

Supplement: Figure 2—source data 2. [file elife-103413-fig2-data2.zip › Figure 2–Source Data 2/ICAM-1.tif]

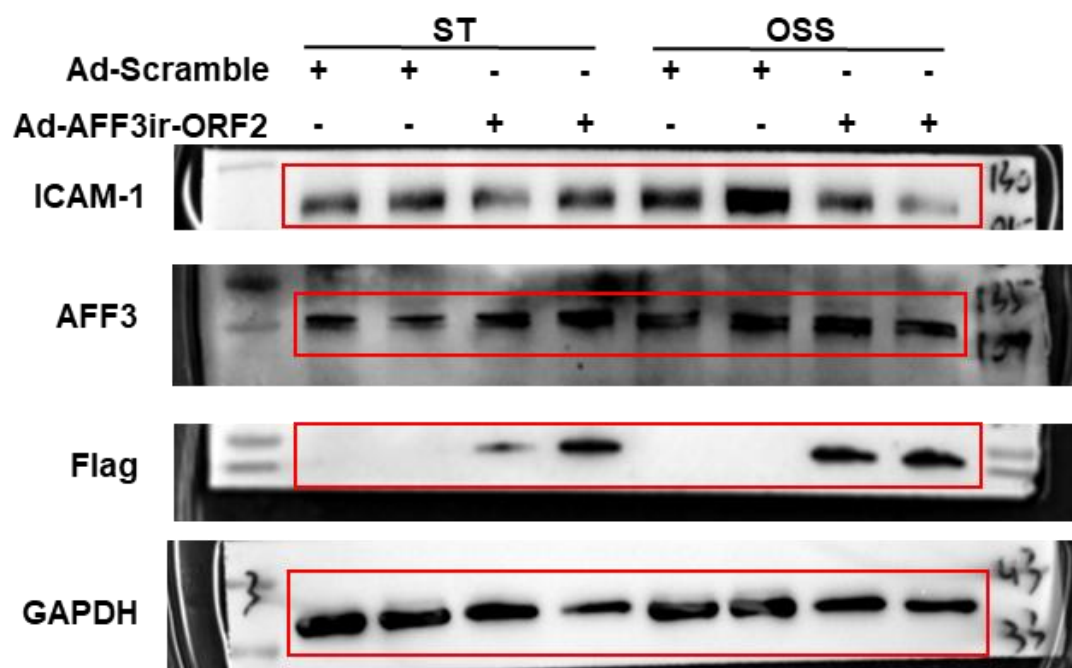

Supplement: Figure 2—source data 3. [file elife-103413-fig2-data3.zip › Figure 2–Source Data 3.pdf]

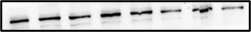

Supplement: Figure 2—source data 4. [file elife-103413-fig2-data4.zip › Figure 2–Source Data 4/AFF3.tif]

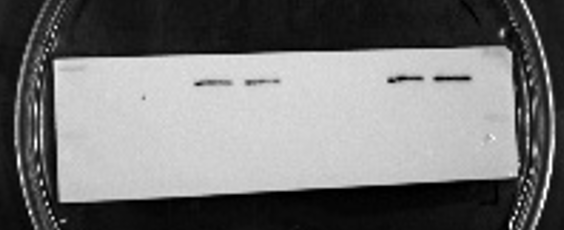

Supplement: Figure 2—source data 4. [file elife-103413-fig2-data4.zip › Figure 2–Source Data 4/FLAG.tif]

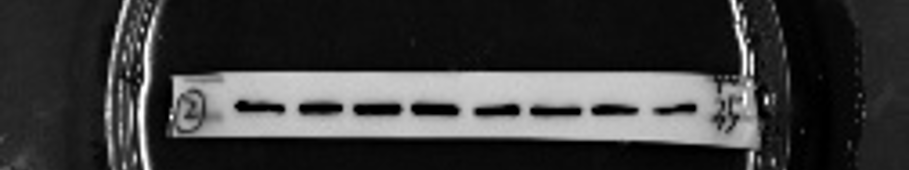

Supplement: Figure 2—source data 4. [file elife-103413-fig2-data4.zip › Figure 2–Source Data 4/GAP.tif]

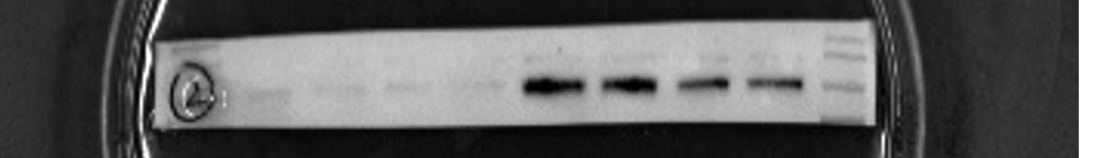

Supplement: Figure 2—source data 4. [file elife-103413-fig2-data4.zip › Figure 2–Source Data 4/ICAM-1.tif]

|                | ST |   |   |   | OSS |   |   |   |
|----------------|----|---|---|---|-----|---|---|---|
| Ad-Scramble    | +  | + | - | - | +   | + | - | - |
| Ad-AFF3ir-ORF2 | -  | - | + | + | -   | - | + | + |

ICAM-1

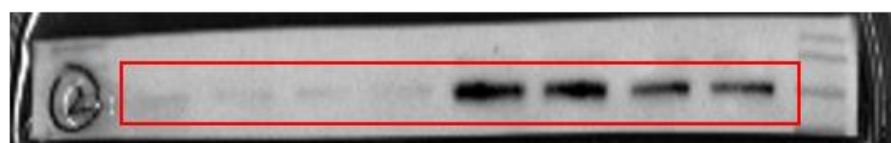

AFF3

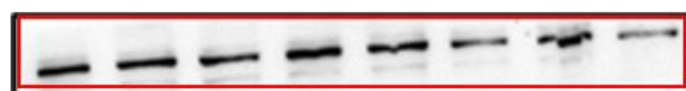

Flag

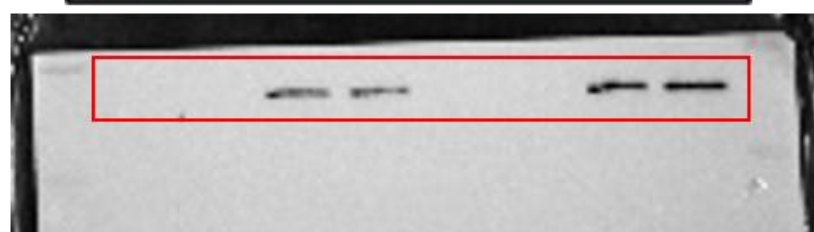

GAPDH

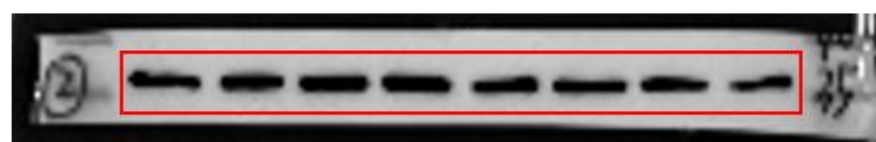

Supplement: Figure 2—source data 5. [file elife-103413-fig2-data5.zip › Figure 2–Source Data 5.pdf]

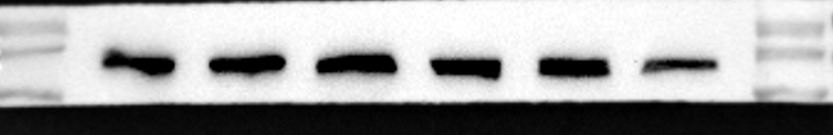

Supplement: Figure 3—figure supplement 1—source data 2. [file elife-103413-fig3-figsupp1-data2.zip › Figure 3–Figure Supplement 1–Source Data 2/AFF3.tif]

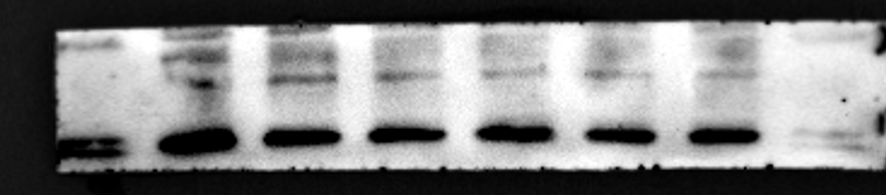

Supplement: Figure 3—figure supplement 1—source data 2. [file elife-103413-fig3-figsupp1-data2.zip › Figure 3–Figure Supplement 1–Source Data 2/AFF3ir-ORF1.tif]

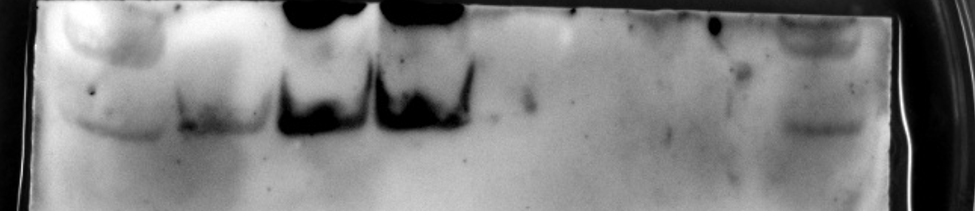

Supplement: Figure 3—figure supplement 1—source data 2. [file elife-103413-fig3-figsupp1-data2.zip › Figure 3–Figure Supplement 1–Source Data 2/AFF3ir-ORF2.tif]

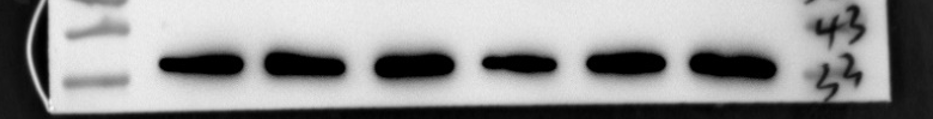

Supplement: Figure 3—figure supplement 1—source data 2. [file elife-103413-fig3-figsupp1-data2.zip › Figure 3–Figure Supplement 1–Source Data 2/GAP.tif]

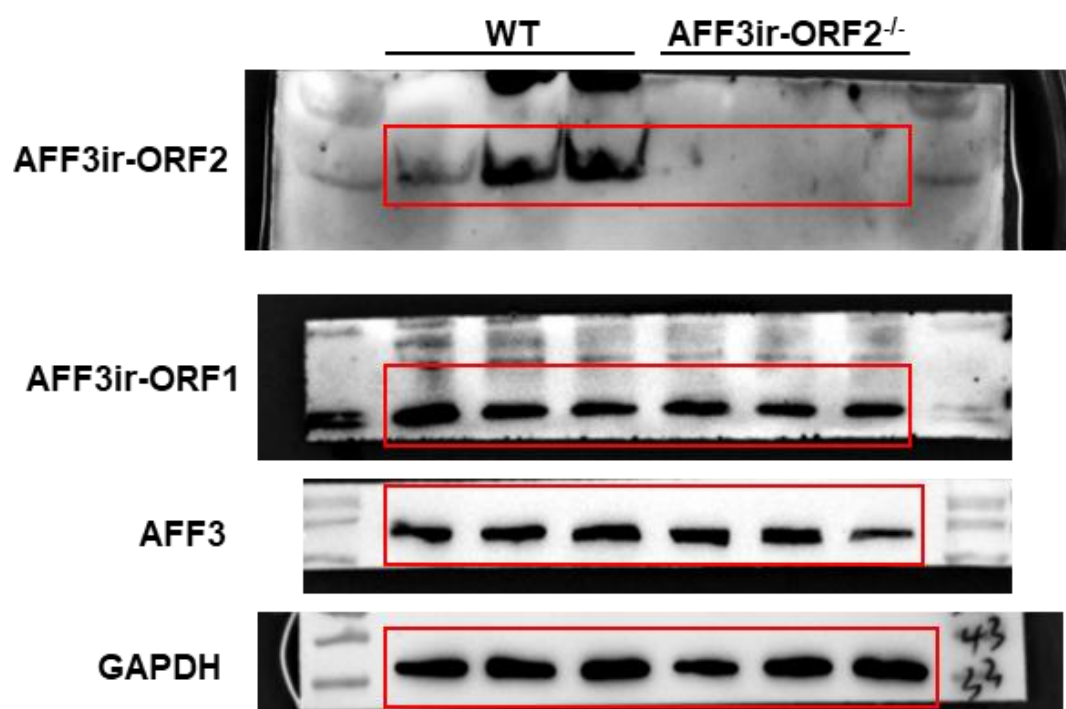

Supplement: Figure 3—figure supplement 1—source data 3. [file elife-103413-fig3-figsupp1-data3.zip › Figure 3–Figure Supplement 1–Source Data 3.pdf]

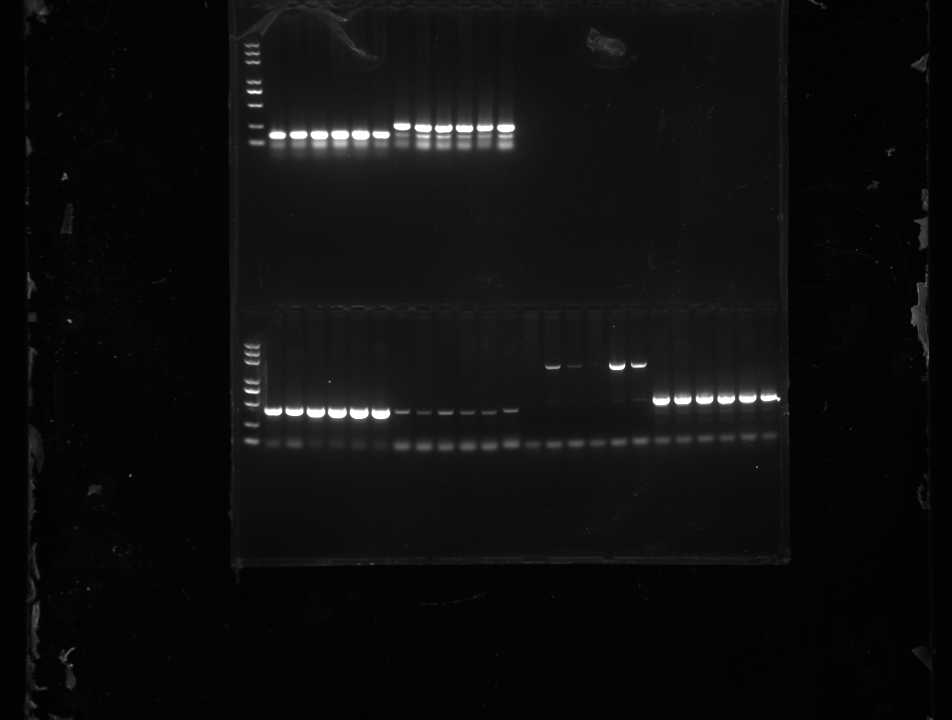

Supplement: Figure 3—figure supplement 1—source data 4. [file elife-103413-fig3-figsupp1-data4.zip › Figure 3–Figure Supplement 1–Source Data 4/FS3-1.tif]

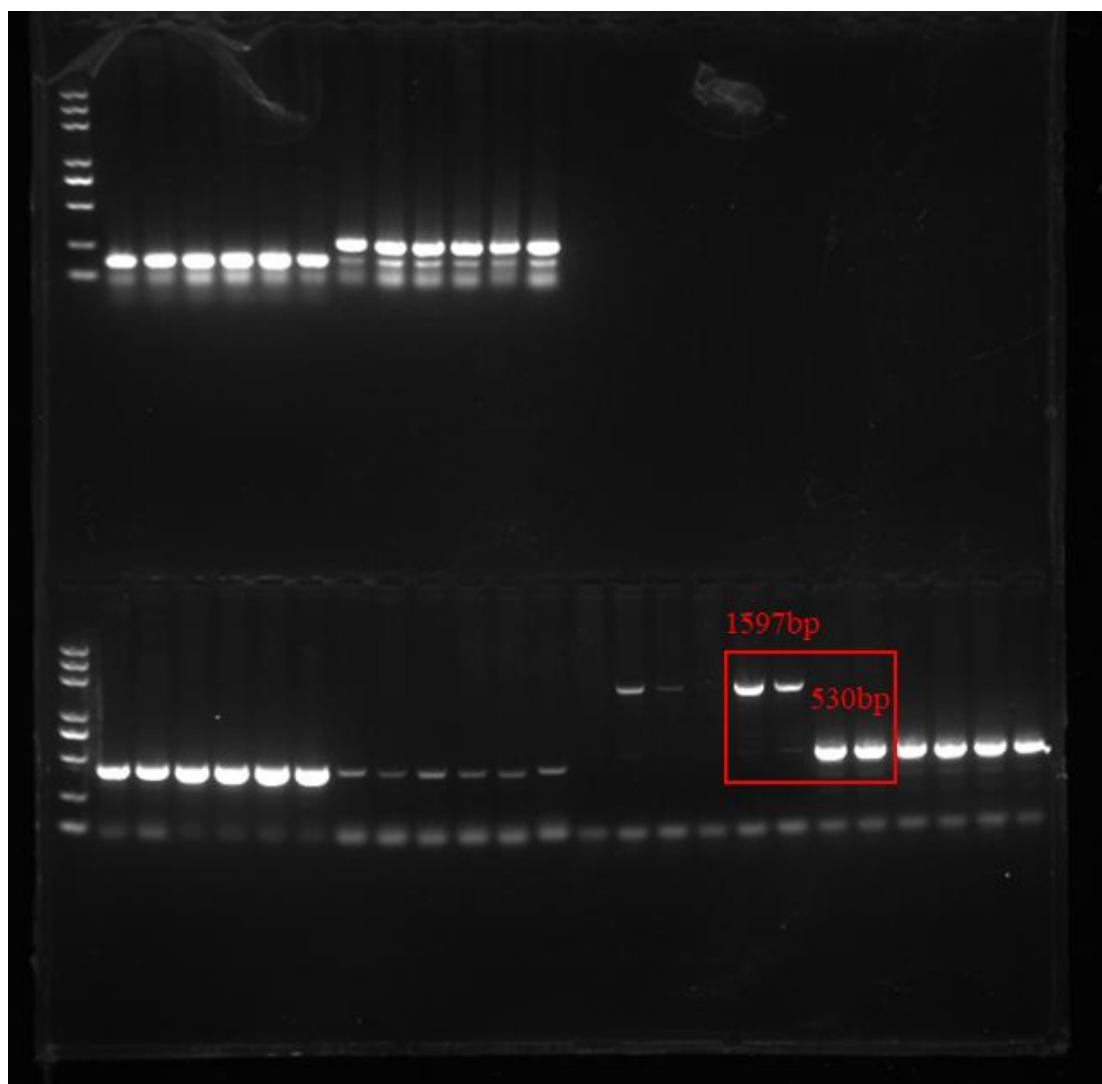

Supplement: Figure 3—figure supplement 1—source data 5. [file elife-103413-fig3-figsupp1-data5.zip › Figure 3–Figure Supplement 1–Source Data 5.pdf]

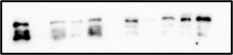

Supplement: Figure 4—source data 2. [file elife-103413-fig4-data2.zip › Figure 4–Source Data 2/AFF3ir-ORF2-IRF8.tif]

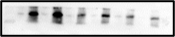

Supplement: Figure 4—source data 2. [file elife-103413-fig4-data2.zip › Figure 4–Source Data 2/AFF3ir-ORF2.tif]

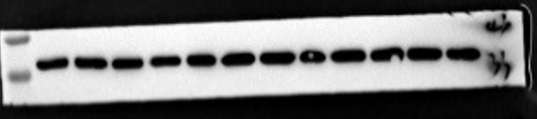

Supplement: Figure 4—source data 2. [file elife-103413-fig4-data2.zip › Figure 4–Source Data 2/GAP-IRF5.tif]

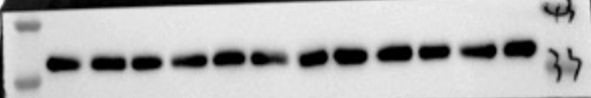

Supplement: Figure 4—source data 2. [file elife-103413-fig4-data2.zip › Figure 4–Source Data 2/GAP-IRF8.tif]

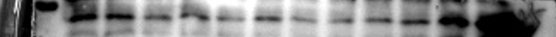

Supplement: Figure 4—source data 2. [file elife-103413-fig4-data2.zip › Figure 4–Source Data 2/IB-AFF3ir-ORF2-IRF5.tif]

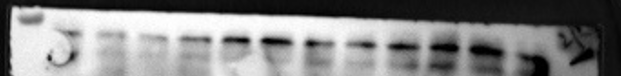

Supplement: Figure 4—source data 2. [file elife-103413-fig4-data2.zip › Figure 4–Source Data 2/IB-AFF3ir-ORF2-IRF8.tif]

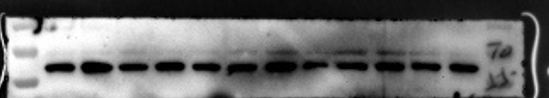

Supplement: Figure 4—source data 2. [file elife-103413-fig4-data2.zip › Figure 4–Source Data 2/IB-IRF5.tif]

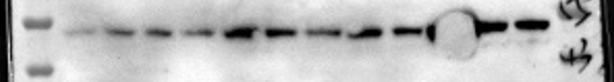

Supplement: Figure 4—source data 2. [file elife-103413-fig4-data2.zip › Figure 4–Source Data 2/IB-IRF8.tif]

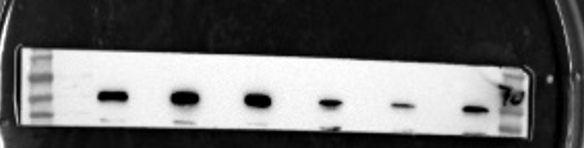

Supplement: Figure 4—source data 2. [file elife-103413-fig4-data2.zip › Figure 4–Source Data 2/IRF5.tif]

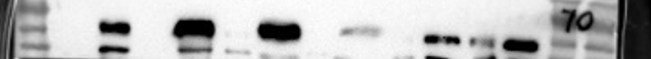

Supplement: Figure 4—source data 2. [file elife-103413-fig4-data2.zip › Figure 4–Source Data 2/IRF8.tif]

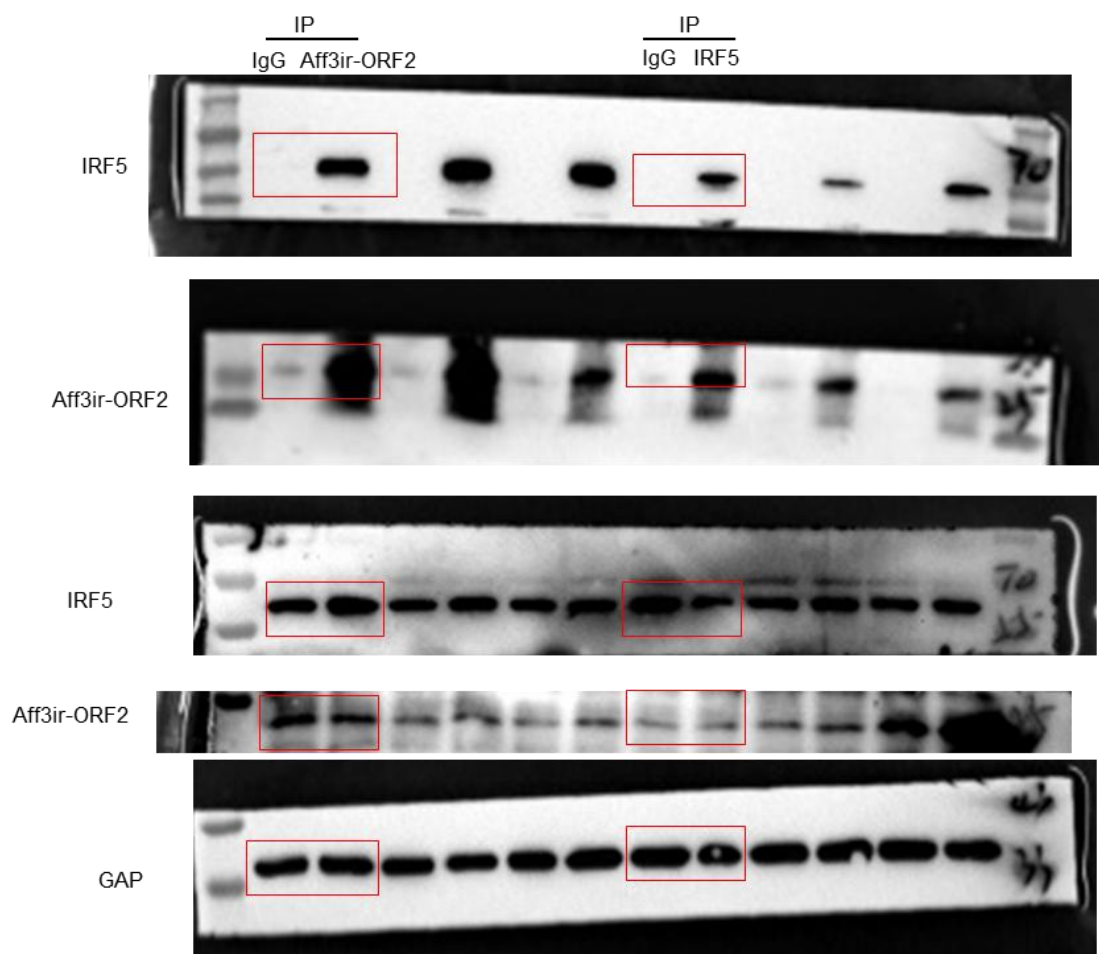

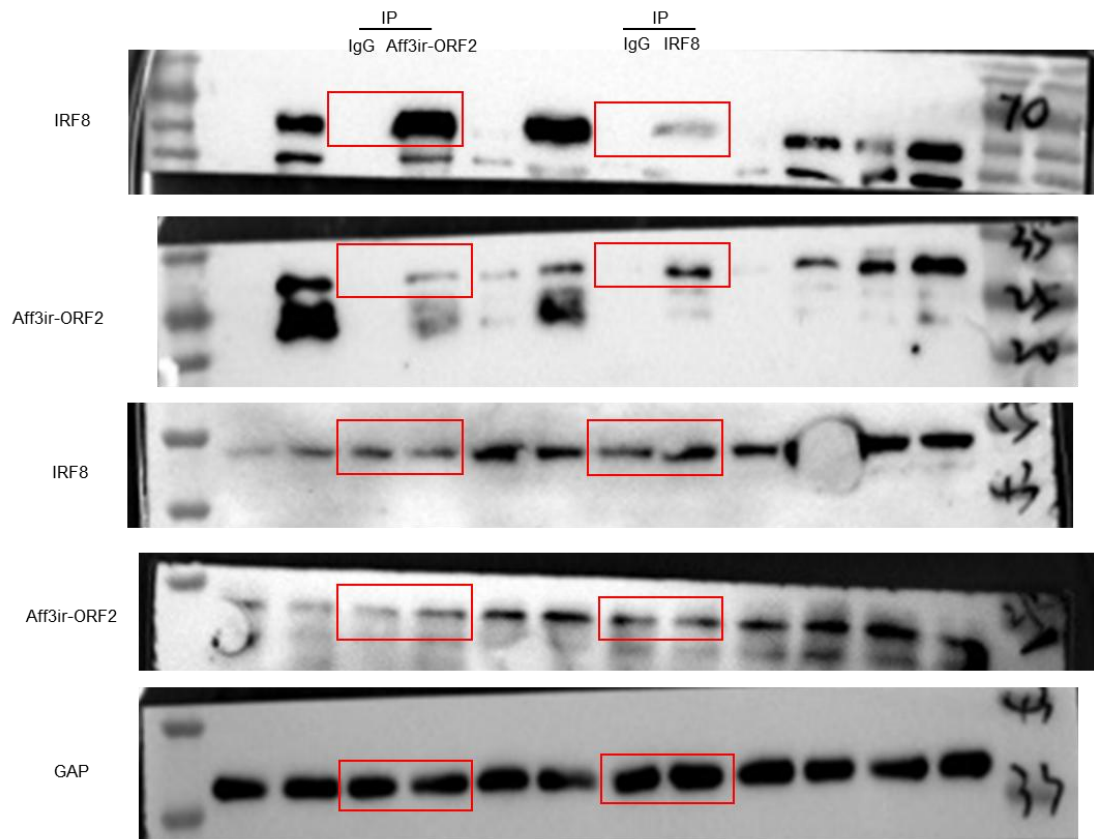

Supplement: Figure 4—source data 3. [file elife-103413-fig4-data3.zip › Figure 4–Source Data 3.pdf]

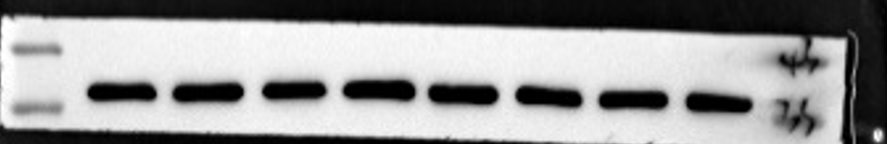

Supplement: Figure 4—source data 4. [file elife-103413-fig4-data4.zip › Figure 4–Source Data 4/gap.tif]

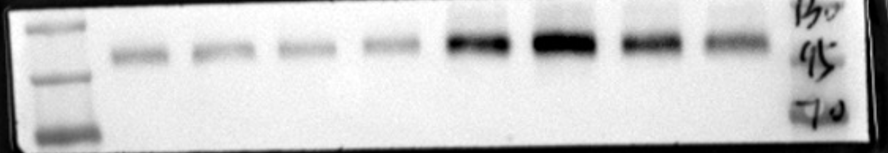

Supplement: Figure 4—source data 4. [file elife-103413-fig4-data4.zip › Figure 4–Source Data 4/icam-1.tif]

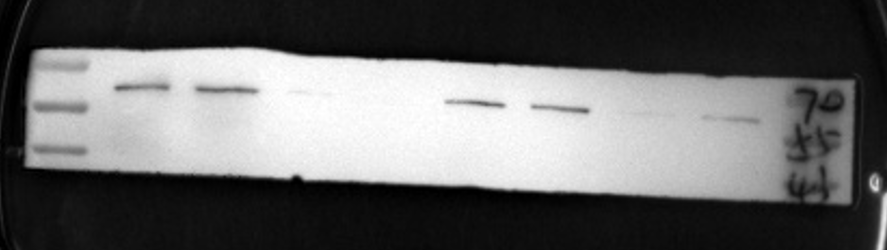

Supplement: Figure 4—source data 4. [file elife-103413-fig4-data4.zip › Figure 4–Source Data 4/irf5.tif]

|                     | ST |   |   |   | OSS |   |   |   |
|---------------------|----|---|---|---|-----|---|---|---|
| ORF2 <sup>-/-</sup> | -  | + | - | + | -   | + | - | + |
| siNC                | +  | + | - | - | +   | + | - | - |
| siIRF5              | -  | - | + | + | -   | - | + | + |

ICAM-1

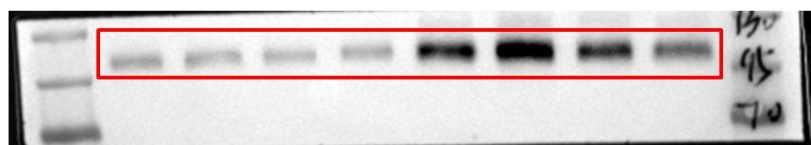

IRF5

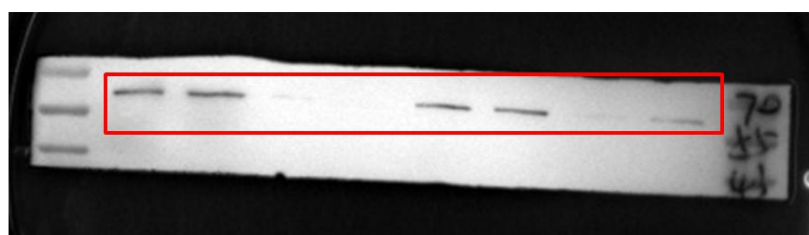

GAP

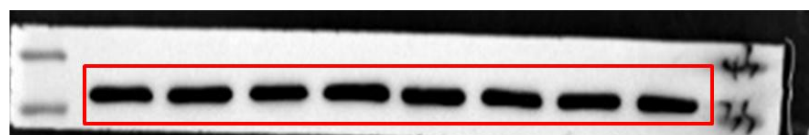

Supplement: Figure 4—source data 5. [file elife-103413-fig4-data5.zip › Figure 4–Source Data 5.pdf]

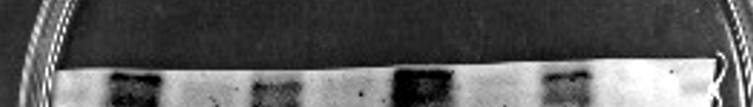

Supplement: Figure 4—source data 6. [file elife-103413-fig4-data6.zip › Figure 4–Source Data 6/AFF3ir-ORF2.tif]

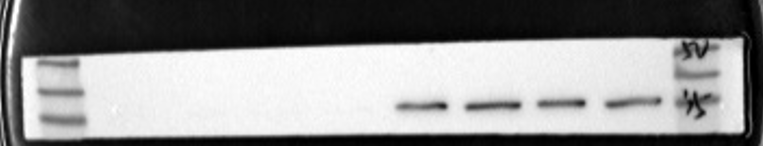

Supplement: Figure 4—source data 6. [file elife-103413-fig4-data6.zip › Figure 4–Source Data 6/GAP.tif]

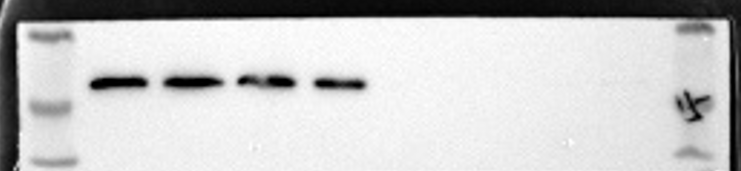

Supplement: Figure 4—source data 6. [file elife-103413-fig4-data6.zip › Figure 4–Source Data 6/Histone H3.tif]

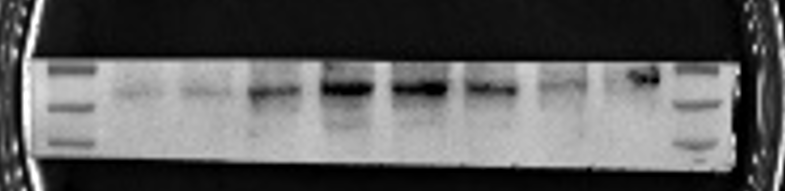

Supplement: Figure 4—source data 6. [file elife-103413-fig4-data6.zip › Figure 4–Source Data 6/IRF5.tif]

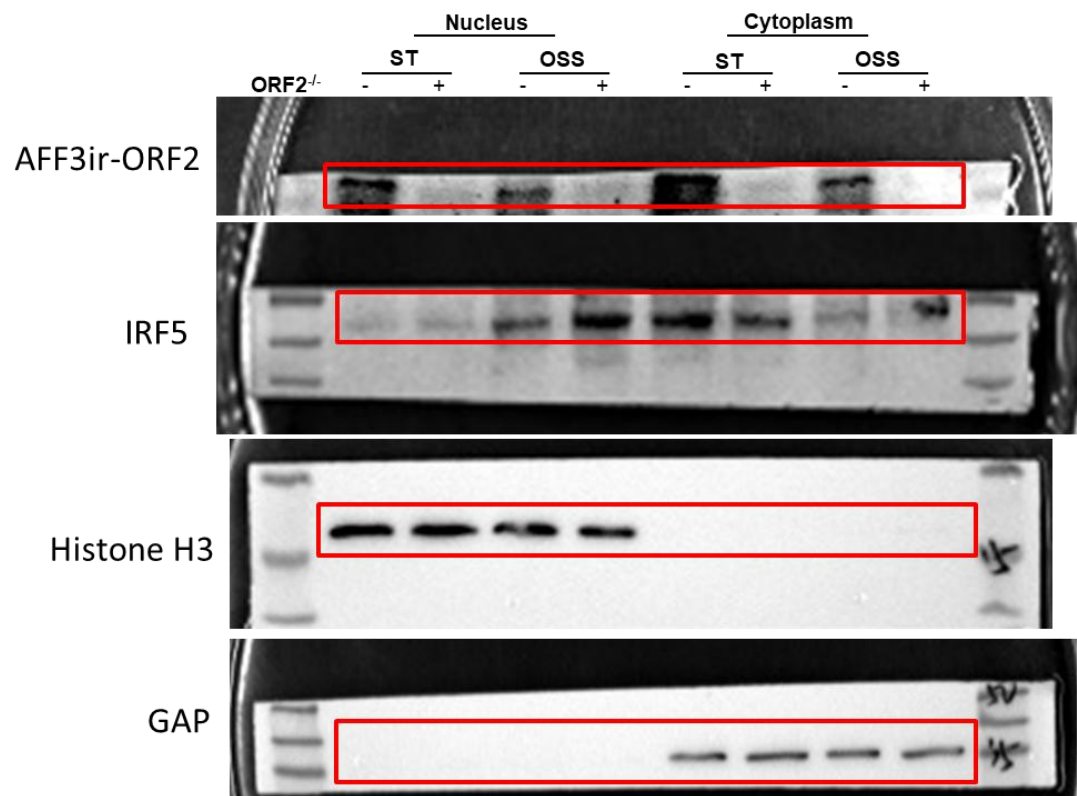

Supplement: Figure 4—source data 7. [file elife-103413-fig4-data7.zip › Figure 4–Source Data 7.pdf]
