# Supplementary material for: Disruption of the novel nested gene Aff3ir mediates disturbed flow-induced atherosclerosis in mice
Source: eLife. 2025 May 2;13:RP103413. doi: 10.7554/eLife.103413 (PMC12048156; doi:10.7554/eLife.103413)
Supplement: Supplementary file 1. [file elife-103413-supp1.docx]

**The sequences of siRNAs.**

| siRNA | Stand | Sequence |
| --- | --- | --- |
| si*Irf5*-1 | \| Forward \| \| --- \| \| Reverse \| | \| GGAAAAGAAACUCUUCUAUTT \| \| --- \| \| AUAGAAGAGUUUCUUUUCCTT \| |
| si*Irf5*-2 | \| Forward \| \| --- \| \| Reverse \| | \| CACCUUUUGAGAUCUUCUUTT \| \| --- \| \| AAGAAGAUCUCAAAAGGUGTT \| |
| si*Irf8*-1 | \| Forward \| \| --- \| \| Reverse \| | \| GUUUAAAGAGGGAGACAAATT \| \| --- \| \| UUUGUCUCCCUCUUUAAACTT \| |
| si*Irf8*-2 | \| Forward \| \| --- \| \| Reverse \| | \| CCAACAAGCUGGAGCGGGATT \| \| --- \| \| UCCCGCUCCAGCUUGUUGGTT \| |
| si*Irf8*-3 | \| Forward \| \| --- \| \| Reverse \| | \| GAGCGAAGUUCCUGAGAUGTT \| \| --- \| \| CAUCUCAGGAACUUCGCUCTT \| |
