# Supplementary material for: Disruption of the novel nested gene Aff3ir mediates disturbed flow-induced atherosclerosis in mice
Source: eLife. 2025 May 2;13:RP103413. doi: 10.7554/eLife.103413 (PMC12048156; doi:10.7554/eLife.103413)
Supplement: Supplementary file 2. [file elife-103413-supp2.docx]

**Primers for qRT-PCR.**

| Gene | Stand | Sequence |
| --- | --- | --- |
| *Gapdh* | \| Forward \| \| --- \| \| Reverse \| | \| AGGTCGGTGTGAACGGATTTG \| \| --- \| \| TGTAGACCATGTAGTTGAGGTCA \| |
| *Irf5* | \| Forward \| \| --- \| \| Reverse \| | \| AGAGACAGGGAAGTACACTGAAG \| \| --- \| \| TGGAAGTCACGGCTTTTGTTAAG \| |
| *Irf8* | \| Forward \| \| --- \| \| Reverse \| | \| CGGGGCTGATCTGGGAAAAT \| \| --- \| \| CACAGCGTAACCTCGTCTTC \| |
| *Aff3* | \| Forward \| \| --- \| \| Reverse \| | \| TCTGTGGGCTCCATCAAC \| \| --- \| \| GACCTCATCGCTGTCCTTT \| |
| *Aff3ir* | \| Forward \| \| --- \| \| Reverse \| | \| GAAACTGAACACGGGAGG \| \| --- \| \| GAACCTGATGCCTGGAAC \| |
| *Vcam1* | \| Forward \| \| --- \| \| Reverse \| | \| AGTTGGGGATTCGGTTGTTCT \| \| --- \| \| CCCCTCATTCCTTACCACCC \| |
| *Icam1* | \| Forward \| \| --- \| \| Reverse \| | \| GTGATGCTCAGGTATCCATCCA \| \| --- \| \| CACAGTTCTCAAAGCACAGCG \| |
| *Il6* | \| Forward \| \| --- \| \| Reverse \| | \| TAGTCCTTCCTACCCCAATTTCC \| \| --- \| \| TTGGTCCTTAGCCACTCCTTC \| |
| *Il1b* | \| Forward \| \| --- \| \| Reverse \| | \| GCAACTGTTCCTGAACTCAACT \| \| --- \| \| ATCTTTTGGGGTCCGTCAACT \| |
